# *N* ^2^-(2-Pyrid­yl)-*N* ^6^-(4-pyrid­yl)pyridine-2,6-diamine

**DOI:** 10.1107/S1600536809033480

**Published:** 2009-08-29

**Authors:** Zhi-Min Wang, Bo Shao

**Affiliations:** a College of Biology and Environmental Engineering, Zhejiang Shuren University, 310015 Hangzhou, People’s Republic of China

## Abstract

In the title compound, C_15_H_13_N_5_, the dihedral angles between the central aromatic ring and and the two peripheral rings are 1.5 (6) and 33.1 (4)°. In the crystal, inter­molecular N—H⋯N hydrogen bonds connect the mol­ecules into a zigzag chain propagating in [100].

## Related literature

For a related structure, see: Huang *et al.* (2004 ▶). For background to metal-organic framework complexes with polypyridylamine ligands, see: Peng *et al.* (2000 ▶); Fang *et al.* (2005 ▶).
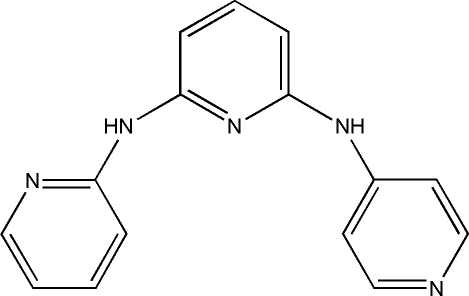


## Experimental

### 

#### Crystal data

C_15_H_13_N_5_
*M* *_r_* = 263.30
Orthorhombic, 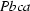

*a* = 11.4884 (15) Å
*b* = 7.3445 (10) Å
*c* = 30.718 (4) Å
*V* = 2591.9 (6) Å^3^
*Z* = 8
Mo *K*α radiation
μ = 0.09 mm^−1^
*T* = 298 K
0.19 × 0.15 × 0.11 mm

#### Data collection

Bruker APEXII CCD diffractometer
Absorption correction: multi-scan (*SADABS*; Bruker, 2005 ▶) *T*_min_ = 0.984, *T*_max_ = 0.991
11972 measured reflections
2304 independent reflections
1529 reflections with *I* > 2σ(*I*)
*R*_int_ = 0.053

#### Refinement

*R*[*F* ^2^ > 2σ(*F* ^2^)] = 0.050
*wR*(*F* ^2^) = 0.187
*S* = 0.82
2304 reflections
182 parameters
1 restraint
H-atom parameters constrained
Δρ_max_ = 0.23 e Å^−3^
Δρ_min_ = −0.15 e Å^−3^

### 

Data collection: *APEX2* (Bruker, 2005 ▶); cell refinement: *SAINT* (Bruker, 2005 ▶); data reduction: *SAINT*; program(s) used to solve structure: *SHELXS97* (Sheldrick, 2008 ▶); program(s) used to refine structure: *SHELXL97* (Sheldrick, 2008 ▶); molecular graphics: *ORTEPIII* (Burnett & Johnson, 1996 ▶), *ORTEP-3 for Windows* (Farrugia, 1997 ▶) and *PLATON* (Spek, 2009 ▶); software used to prepare material for publication: *SHELXL97*.

## Supplementary Material

Crystal structure: contains datablocks I, global. DOI: 10.1107/S1600536809033480/hb5059sup1.cif

Structure factors: contains datablocks I. DOI: 10.1107/S1600536809033480/hb5059Isup2.hkl

Additional supplementary materials:  crystallographic information; 3D view; checkCIF report

## Figures and Tables

**Table 1:** Hydrogen-bond geometry (Å, °)

*D*—H⋯*A*	*D*—H	H⋯*A*	*D*⋯*A*	*D*—H⋯*A*
N4—H4*A*⋯N1^i^	0.86	2.22	3.038 (3)	160
N2—H2*A*⋯N3^ii^	0.86	2.35	3.198 (3)	170

Symmetry codes: (i) 
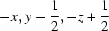
; (ii) 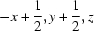.

**Fig. 1.**
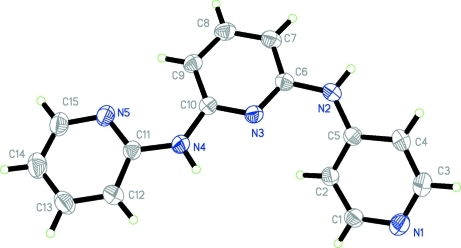
The molecular structure of (I) Ellipsoids are drawn at the the 30% probability level. H atoms are shown as spheres of arbitrary radius.

**Fig. 2.**
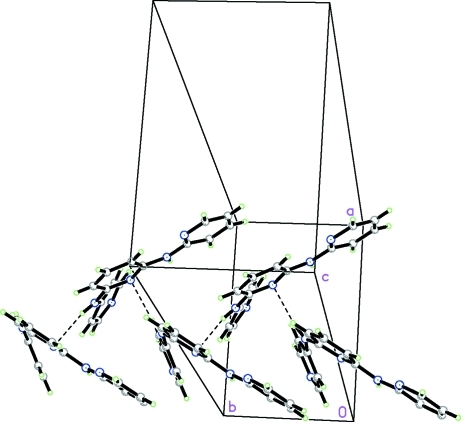
Partial packing of (I) view showing the formation of a chain through N-H···N hydrogen bonds. Hydrogen bonds are shown as dashed lines.

## supplementary crystallographic information

### Comment

Metal-organic frameworks complexes with polypyridylamine ligands, bearing diverse networks and special optical and electromagnetic properties (Peng *et al.*,  2000), have aroused great interest among researchers. Tri-pyridyldiamine ligand usually exhibits donor as well as acceptor properties and  can be used as a popular chelating ligand (Fang *et al.*, 2005). The crystals of the title compound were obatined unintentionally as the harvested product of the mild reaction of N^2^-(pydidin-2-yl)-N^6^-(pydidin-4-yl)pyridine-2,6-diamine, zinc salt.

The molecular structure of the title compound is shown in Fig. 1. In the crystal structure, intermoelcular N-H···N hydorgen bonds connect molecules into one-dimensional chain along a axis (Table 1), as shown in Figure 2. The three pyridine rings of the title compound are not coplanar. The dihedral angles between the planes of the central pyridine ring and two peripheral rings are 1.5 (6) and 146.9 (4)° respectively, which is very different from the Cd complex with [2,6-bis(2-pyridylamino)pyridine] [15.6 (5) and 34.1 (3)°] (Huang *et al.*,  2004).

### Experimental

N^2^-(pydidin-2-yl)-N^6^-(pydidin-4-yl)pyridine-2,6-diamine (0.27 mg,0.1 mmol), Zn(CH_3_COO)_2_ (0.43 mg, 0.1 mmol), were added to dry ethanol. The mixture was heated and stirred for six hours under reflux. The resultant was then filtered off to give a pure solution which was treated by diethyl ether in a closed vessel. Two weeks later, colourless blocks of (I) were obtained.

### Refinement

The H atoms were positioned geometrically and treated as riding on their parent atoms, with C—H distances of 0.93Å (pyridine ring), N—H = 0.86 Å (amine group), and with U_iso_(H) 1.2U_eq_(carrier).

### Crystal data

**Table:**

C_15_H_13_N_5_	*F*(000) = 1104
*M**_r_* = 263.30	*D*_x_ = 1.350 Mg m^−^^3^
Orthorhombic, *P**b**c**a*	Mo *K*α radiation, λ = 0.71073 Å
Hall symbol: -P 2ac 2ab	Cell parameters from 2304 reflections
*a* = 11.4884 (15) Å	θ = 2.2–25.2°
*b* = 7.3445 (10) Å	µ = 0.09 mm^−^^1^
*c* = 30.718 (4) Å	*T* = 298 K
*V* = 2591.9 (6) Å^3^	Block, colourless
*Z* = 8	0.19 × 0.15 × 0.11 mm

### Data collection

**Table:**

Bruker APEXII CCD diffractometer	2304 independent reflections
Radiation source: fine-focus sealed tube	1529 reflections with *I* > 2σ(*I*)
graphite	*R*_int_ = 0.053
φ and ω scans	θ_max_ = 25.2°, θ_min_ = 2.2°
Absorption correction: multi-scan (*SADABS*; Bruker, 2005)	*h* = −13→11
*T*_min_ = 0.984, *T*_max_ = 0.991	*k* = −8→8
11972 measured reflections	*l* = −34→36

### Refinement

**Table:**

Refinement on *F*^2^	Secondary atom site location: difference Fourier map
Least-squares matrix: full	Hydrogen site location: inferred from neighbouring sites
*R*[*F*^2^ > 2σ(*F*^2^)] = 0.050	H-atom parameters constrained
*wR*(*F*^2^) = 0.187	*w* = 1/[σ^2^(*F*_o_^2^) + (0.158*P*)^2^ + 0.03*P*] where *P* = (*F*_o_^2^ + 2*F*_c_^2^)/3
*S* = 0.82	(Δ/σ)_max_ < 0.001
2304 reflections	Δρ_max_ = 0.23 e Å^−^^3^
182 parameters	Δρ_min_ = −0.15 e Å^−^^3^
1 restraint	Extinction correction: *SHELXL97* (Sheldrick, 2008), Fc^*^=kFc[1+0.001xFc^2^λ^3^/sin(2θ)]^-1/4^
Primary atom site location: structure-invariant direct methods	Extinction coefficient: 0.011 (2)

### Special details

**Table:**

Geometry. All esds (except the esd in the dihedral angle between two l.s. planes) are estimated using the full covariance matrix. The cell esds are taken into account individually in the estimation of esds in distances, angles and torsion angles; correlations between esds in cell parameters are only used when they are defined by crystal symmetry. An approximate (isotropic) treatment of cell esds is used for estimating esds involving l.s. planes.
Refinement. Refinement of F^2^ against ALL reflections. The weighted R-factor wR and goodness of fit S are based on F^2^, conventional R-factors R are based on F, with F set to zero for negative F^2^. The threshold expression of F^2^ > 2sigma(F^2^) is used only for calculating R-factors(gt) etc. and is not relevant to the choice of reflections for refinement. R-factors based on F^2^ are statistically about twice as large as those based on F, and R- factors based on ALL data will be even larger.

### Fractional atomic coordinates and isotropic or equivalent isotropic displacement parameters (Å^2^)

**Table:**

	*x*	*y*	*z*	*U*_iso_*/*U*_eq_	
N3	0.09096 (15)	1.0142 (2)	0.36725 (5)	0.0482 (5)	
C11	−0.05825 (17)	0.6096 (3)	0.40354 (7)	0.0485 (6)	
N5	−0.06267 (16)	0.6024 (3)	0.44682 (6)	0.0573 (6)	
N2	0.18582 (17)	1.2747 (2)	0.34296 (6)	0.0586 (6)	
H2A	0.2400	1.3509	0.3493	0.070*	
C1	0.0235 (2)	1.1978 (3)	0.24144 (7)	0.0544 (6)	
H1	−0.0457	1.1441	0.2324	0.065*	
C9	0.06292 (18)	0.9532 (3)	0.44324 (7)	0.0520 (6)	
H9	0.0349	0.8779	0.4652	0.062*	
C6	0.14379 (19)	1.1700 (3)	0.37742 (7)	0.0482 (6)	
C5	0.15115 (19)	1.2712 (3)	0.29979 (7)	0.0476 (6)	
C4	0.22262 (19)	1.3511 (3)	0.26852 (8)	0.0557 (6)	
H4	0.2920	1.4068	0.2766	0.067*	
N4	−0.00874 (17)	0.7580 (2)	0.38310 (6)	0.0553 (6)	
H4A	−0.0140	0.7558	0.3552	0.066*	
C2	0.04739 (18)	1.1955 (3)	0.28529 (7)	0.0499 (6)	
H2	−0.0049	1.1443	0.3049	0.060*	
N1	0.09143 (19)	1.2706 (3)	0.21080 (6)	0.0623 (6)	
C7	0.1620 (2)	1.2251 (3)	0.42019 (7)	0.0570 (7)	
H7	0.2001	1.3335	0.4266	0.068*	
C10	0.04771 (17)	0.9094 (3)	0.39941 (7)	0.0450 (5)	
C8	0.1212 (2)	1.1128 (3)	0.45271 (7)	0.0595 (7)	
H8	0.1332	1.1451	0.4816	0.071*	
C12	−0.1031 (2)	0.4715 (3)	0.37727 (8)	0.0612 (6)	
H12	−0.0978	0.4792	0.3471	0.073*	
C3	0.1897 (2)	1.3467 (3)	0.22582 (8)	0.0638 (7)	
H3	0.2391	1.4007	0.2056	0.077*	
C15	−0.1133 (2)	0.4549 (4)	0.46451 (9)	0.0694 (8)	
H15	−0.1165	0.4479	0.4947	0.083*	
C14	−0.1602 (2)	0.3157 (4)	0.44141 (10)	0.0759 (8)	
H14	−0.1947	0.2172	0.4554	0.091*	
C13	−0.1552 (2)	0.3242 (4)	0.39659 (10)	0.0737 (8)	
H13	−0.1867	0.2313	0.3797	0.088*	

### Atomic displacement parameters (Å^2^)

**Table:**

	*U*^11^	*U*^22^	*U*^33^	*U*^12^	*U*^13^	*U*^23^
N3	0.0552 (11)	0.0459 (11)	0.0436 (10)	−0.0016 (8)	−0.0036 (8)	−0.0010 (8)
C11	0.0482 (12)	0.0477 (13)	0.0495 (13)	0.0023 (10)	−0.0020 (9)	0.0048 (10)
N5	0.0608 (12)	0.0589 (13)	0.0521 (12)	−0.0004 (10)	0.0003 (9)	0.0112 (9)
N2	0.0616 (12)	0.0579 (12)	0.0561 (12)	−0.0192 (9)	−0.0075 (9)	0.0004 (9)
C1	0.0567 (13)	0.0496 (13)	0.0571 (15)	−0.0028 (11)	−0.0072 (11)	0.0020 (10)
C9	0.0560 (13)	0.0574 (14)	0.0426 (12)	0.0005 (11)	0.0012 (10)	−0.0015 (10)
C6	0.0480 (12)	0.0466 (12)	0.0500 (13)	0.0002 (10)	−0.0023 (9)	−0.0022 (10)
C5	0.0552 (13)	0.0387 (11)	0.0488 (13)	0.0003 (9)	−0.0001 (10)	−0.0028 (9)
C4	0.0539 (13)	0.0468 (13)	0.0664 (16)	−0.0066 (10)	0.0032 (11)	0.0029 (10)
N4	0.0754 (14)	0.0498 (12)	0.0408 (10)	−0.0103 (10)	−0.0013 (8)	0.0004 (7)
C2	0.0501 (13)	0.0484 (12)	0.0513 (13)	−0.0029 (10)	0.0002 (10)	0.0033 (10)
N1	0.0756 (13)	0.0584 (13)	0.0530 (12)	0.0035 (10)	0.0027 (10)	0.0051 (9)
C7	0.0561 (14)	0.0582 (15)	0.0568 (15)	−0.0068 (11)	−0.0035 (11)	−0.0111 (11)
C10	0.0455 (12)	0.0447 (12)	0.0449 (12)	0.0029 (9)	0.0004 (9)	−0.0003 (9)
C8	0.0621 (15)	0.0700 (17)	0.0465 (13)	−0.0007 (13)	−0.0007 (10)	−0.0125 (11)
C12	0.0626 (15)	0.0582 (15)	0.0628 (14)	−0.0089 (12)	−0.0073 (11)	−0.0010 (12)
C3	0.0725 (15)	0.0593 (16)	0.0596 (15)	0.0017 (12)	0.0130 (12)	0.0081 (11)
C15	0.0624 (16)	0.0743 (18)	0.0715 (17)	−0.0059 (14)	0.0016 (12)	0.0216 (14)
C14	0.0604 (16)	0.0711 (18)	0.096 (2)	−0.0107 (14)	−0.0013 (14)	0.0256 (16)
C13	0.0691 (17)	0.0592 (16)	0.093 (2)	−0.0180 (13)	−0.0082 (15)	0.0030 (14)

### Geometric parameters (Å, °)

**Table:**

N3—C6	1.332 (3)	C4—C3	1.366 (3)
N3—C10	1.347 (3)	C4—H4	0.9300
C11—N5	1.331 (3)	N4—C10	1.381 (2)
C11—N4	1.380 (3)	N4—H4A	0.8600
C11—C12	1.395 (3)	C2—H2	0.9300
N5—C15	1.344 (3)	N1—C3	1.341 (3)
N2—C5	1.385 (3)	C7—C8	1.378 (3)
N2—C6	1.394 (3)	C7—H7	0.9300
N2—H2A	0.8600	C8—H8	0.9300
C1—N1	1.335 (3)	C12—C13	1.371 (3)
C1—C2	1.375 (3)	C12—H12	0.9300
C1—H1	0.9300	C3—H3	0.9300
C9—C8	1.381 (3)	C15—C14	1.356 (4)
C9—C10	1.395 (3)	C15—H15	0.9300
C9—H9	0.9300	C14—C13	1.379 (4)
C6—C7	1.390 (3)	C14—H14	0.9300
C5—C2	1.388 (3)	C13—H13	0.9300
C5—C4	1.393 (3)		
			
C6—N3—C10	119.13 (18)	C1—C2—H2	120.6
N5—C11—N4	120.07 (19)	C5—C2—H2	120.6
N5—C11—C12	122.3 (2)	C1—N1—C3	114.6 (2)
N4—C11—C12	117.6 (2)	C8—C7—C6	117.4 (2)
C11—N5—C15	116.9 (2)	C8—C7—H7	121.3
C5—N2—C6	128.11 (18)	C6—C7—H7	121.3
C5—N2—H2A	115.9	N3—C10—N4	111.54 (17)
C6—N2—H2A	115.9	N3—C10—C9	121.97 (19)
N1—C1—C2	125.4 (2)	N4—C10—C9	126.5 (2)
N1—C1—H1	117.3	C7—C8—C9	121.3 (2)
C2—C1—H1	117.3	C7—C8—H8	119.3
C8—C9—C10	117.4 (2)	C9—C8—H8	119.3
C8—C9—H9	121.3	C13—C12—C11	119.0 (2)
C10—C9—H9	121.3	C13—C12—H12	120.5
N3—C6—C7	122.7 (2)	C11—C12—H12	120.5
N3—C6—N2	116.95 (19)	N1—C3—C4	125.0 (2)
C7—C6—N2	120.3 (2)	N1—C3—H3	117.5
N2—C5—C2	124.1 (2)	C4—C3—H3	117.5
N2—C5—C4	118.9 (2)	N5—C15—C14	124.6 (3)
C2—C5—C4	117.0 (2)	N5—C15—H15	117.7
C3—C4—C5	119.3 (2)	C14—C15—H15	117.7
C3—C4—H4	120.4	C15—C14—C13	118.2 (2)
C5—C4—H4	120.4	C15—C14—H14	120.9
C11—N4—C10	131.6 (2)	C13—C14—H14	120.9
C11—N4—H4A	114.2	C12—C13—C14	119.1 (3)
C10—N4—H4A	114.2	C12—C13—H13	120.5
C1—C2—C5	118.7 (2)	C14—C13—H13	120.5

### Hydrogen-bond geometry (Å, °)

**Table:**

*D*—H···*A*	*D*—H	H···*A*	*D*···*A*	*D*—H···*A*
N4—H4A···N1^i^	0.86	2.22	3.038 (3)	160
N2—H2A···N3^ii^	0.86	2.35	3.198 (3)	170

Symmetry codes: (i) −*x*, *y*−1/2, −*z*+1/2; (ii) −*x*+1/2, *y*+1/2, *z*.
